# Mitochondria as Potential Targets in Alzheimer Disease Therapy: An Update

**DOI:** 10.3389/fphar.2019.00902

**Published:** 2019-08-23

**Authors:** Giovanna Cenini, Wolfgang Voos

**Affiliations:** Institut für Biochemie und Molekularbiologie, Rheinische Friedrich-Wilhelms-Universität Bonn, Bonn, Germany

**Keywords:** Alzheimer disease, therapeutic strategy, mitochondria, mitochondrial dysfunction, mitochondrial therapy

## Abstract

Alzheimer disease (AD) is a progressive and deleterious neurodegenerative disorder that affects mostly the elderly population. At the moment, no effective treatments are available in the market, making the whole situation a compelling challenge for societies worldwide. Recently, novel mechanisms have been proposed to explain the etiology of this disease leading to the new concept that AD is a multifactor pathology. Among others, the function of mitochondria has been considered as one of the intracellular processes severely compromised in AD since the early stages and likely represents a common feature of many neurodegenerative diseases. Many mitochondrial parameters decline already during the aging, reaching an extensive functional failure concomitant with the onset of neurodegenerative conditions, although the exact timeline of these events is still unclear. Thereby, it is not surprising that mitochondria have been already considered as therapeutic targets in neurodegenerative diseases including AD. Together with an overview of the role of mitochondrial dysfunction, this review examines the pros and cons of the tested therapeutic approaches targeting mitochondria in the context of AD. Since mitochondrial therapies in AD have shown different degrees of progress, it is imperative to perform a detailed analysis of the significance of mitochondrial deterioration in AD and of a pharmacological treatment at this level. This step would be very important for the field, as an effective drug treatment in AD is still missing and new therapeutic concepts are urgently needed.

## Introduction

Alzheimer disease (AD) is a complex and heterogeneous disorder strongly affecting the cognitive functions and the memory of seniors. 

**Figure 1:**
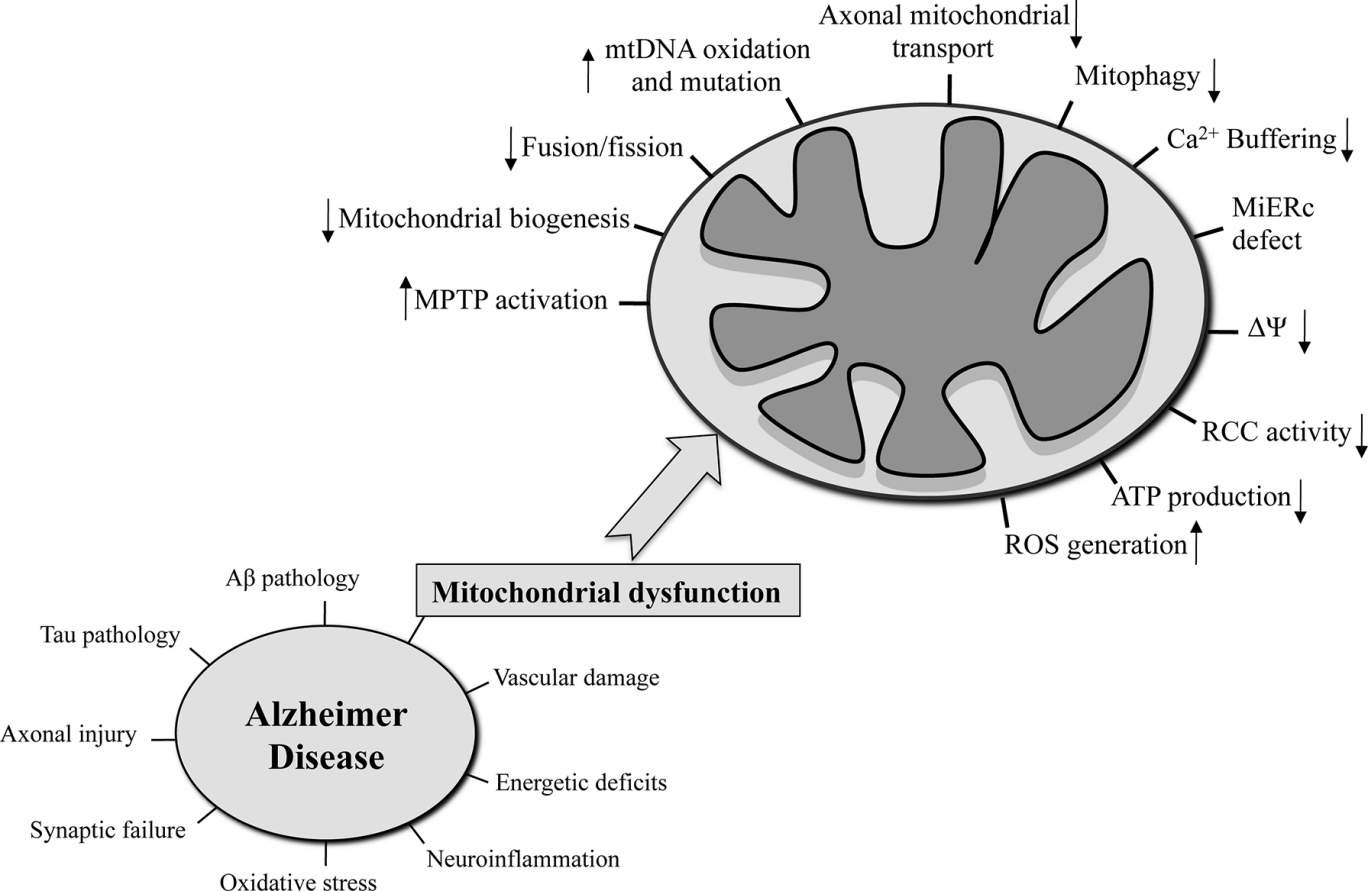
The hallmarks that characterized AD are reported in the left side of the figure. On the right side, the mitochondria-related functions that are seriously compromised in AD are on focus.

Many risk factors were proposed to be significant contributors for the AD onset such as senescence, autophagy defects, genetic factors [i.e., ApolipoproteinaE-allele4 (APOE4), Triggering receptor expressed on myeloid cells 2 (Trem2)], microbiota alterations, lifestyle choices, cardiovascular and traumatic brain injury, as well as environmental factors (level of education, hypertension, obesity, diabetes, smoking, hearing loss, depression, physical inactivity, social isolation) (Livingston et al., 2017). It is now well accepted that important cellular pathways are compromised in AD. Together with intraneuronal neurofibrillary tangles (NFT) made of hyperphosphorylated tau protein and the extraneuronal senile plaques (SP) made of beta-amyloid (Aβ) peptides, synaptic failure, vascular damage, increased oxidative stress, neuronal and axonal injury, microglia-regulated neuroinflammation, and mitochondrial dysfunction are hallmarks of the disease (**Figure 1**).

Along the past years, Aβ peptides have been considered one of the most promising therapeutic targets for AD. However, many clinical studies based on the Aβ cascade hypothesis failed, and the idea that Aβ pathology is not anymore the leading primary cause of AD has risen (Morris et al., 2018). Instead, nowadays the belief that AD is a multi-factorial disease is growing steadily, and mitochondrial dysfunction is one of the factors that may actively contribute to the disease onset and progression (Iturria-Medina et al., 2017; Veitch et al., 2019). Despite that, a logical temporal order of the events in AD, as well as a valid and effective therapy, is still missing. However, our society urgently requires medical interventions to counteract this deleterious disease because of the severe negative impact on the quality of lives of the afflicted patients as well as on the health system as a whole due to a rapidly aging population. 

This review focuses on the description of the role of mitochondrial dysfunction and the status of mitochondrial therapy in AD. The main question addressed here is: could the mitochondrial organelle be a valid pharmacologic target to prevent or delay the AD onset or to block the AD progression?

## Mitochondria

The mitochondrion is a cellular organelle with a characteristic and unique structure formed by two membranes, respectively called outer mitochondrial membrane (OMM) and inner mitochondrial membrane (IMM) that surround the matrix. Mitochondria are defined as the powerhouse of the cell because every cell in the human body relies on the energy provided by these organelles to sustain their vital functions. Mitochondrial energy production via the so-called process of oxidative phosphorylation takes place at the IMM through the activity of respiratory chain complexes (RCC), generating an inner membrane potential (mtΔΨ) that is used by the ATP-synthase enzyme complex to synthesize adenosine triphosphate (ATP). This process depends on the supply of reducing equivalents by the end-oxidation of nutrients *via* the Krebs cycle or β-oxidation in the mitochondrial matrix compartment (Stock et al., 2000). Mitochondria contain their own DNA (mtDNA) located in the matrix that encodes mainly 13 protein subunits of the RCC. All other mitochondrial protein components are encoded in the nuclear DNA (nuDNA) and are imported into the organelle after the translation at cytosolic ribosomes. Hence, the maintenance of an entire and functional mitochondrial proteome requires a fine-tuned and well-coordinated sequence of many reactions and a close integration of organellar and cellular biogenesis processes (Pfanner et al., 2019). 

Neurons are strictly dependent on the presence of mitochondria in particular at the synapses where these organelles produce ATP and buffer Ca^2+^-ion concentration, both fundamental processes for the implementation of neurotransmission and generation of membrane potential along the axon (Li et al., 2004; Verstreken et al., 2005; Gazit et al., 2016). This justifies the high amount of mitochondria at the synaptic area, higher than any other part of the neurons. Linked to that, a correct and efficient transport of neuronal mitochondria at the synaptic terminals is fundamental for their correct function. Both non-synaptic and synaptic mitochondria are usually synthesized in the neuronal soma and then transported in the other area of the neurons where they are required. The transport of mitochondria along the axons is guaranteed *via* microtubules and requires motor proteins such as kinesin, dynein, as well as the OMM protein Mitochondrial Rho GTPase (Miro). Axonal transport of mitochondria is also influenced by the metabolic demand and the Ca^2+^ status at the synaptic level (Yi et al., 2004; Glater et al., 2006; Russo et al., 2009; Sheng and Cai, 2012).

The enzymatic activity of the mitochondrial RCC results essentially in two “side effects.” First, the generation of the mtΔΨ along the IMM is essential also for the execution of mitochondrial import of nuclear-encoded proteins and overall it is a parameter that reflects the health status of mitochondria and cells (Shariff et al., 2004). Second, a leakage of electrons from the RCC contributes significantly to the formation of reactive oxygen species (ROS). Therefore, ROS are considered a typical by-product of bioenergetic pathways (Quinlan et al., 2013). However, under normal physiological conditions, ROS production is well balanced by the presence of adequate antioxidant systems, and the damage to the diverse cellular constituents is contained. However, during aging, as well as during several pathological conditions, in particular in neurodegenerative diseases, this equilibrium becomes unbalanced. Increased ROS concentrations result in molecular damage at the site where they are produced or, through diffusion, in surrounding areas, leading to the generation of the so-called oxidative stress condition. ROS targets essentially comprise all cellular macromolecules, ranging from proteins, lipids, carbohydrates, up to nucleic acids (Cipak Gasparovic et al., 2017). The hippocampus region, the cortex, and more generally the brain are particularly vulnerable to oxidative stress because of their high consumption of oxygen and dependence on mitochondrial energy production. This susceptibility is increased by low levels of antioxidant defenses and a high content of polyunsaturated fats, which are especially vulnerable to oxidative alterations (Cobley et al., 2018).

Mitochondria form a dynamic tubular network extended throughout the cytosol, a behavior that is often misrepresented by the cell biology textbooks. Two crucial processes, fusion and fission, regulate the entire morphology and structure of this mitochondrial network (Mishra and Chan, 2016). During the fission reaction, a part of the mitochondrial tubule is divided into fragments, a process that is regulated by a member of the dynamin family, Dynamin-1-like protein (Drp1), together with the OMM fission factors Mitochondrial fission 1 protein (Fis1) and Mitochondrial dynamics protein MID49 [Mitochondrial elongation factor 2 (MIEF2)]. Fusion, where two or more pieces of mitochondria are fused together to one structure, happens through joint activity of the proteins Dynamin-like 120 kDa protein [or Optic atrophy protein 1 (OPA1)] and Mitofusin 1 and 2 (Mfn1 and Mfn2). Fusion/fission processes together with the precursor proteins import and internal proteins translation are part of the mitochondrial biogenesis in which the cells increase their mitochondrial mass (Sanchis-Gomar et al., 2014). A master regulator of mitochondrial biogenesis is Peroxisome-proliferator-activated receptor γ coactivator-1α (PGC-1α) (Scarpulla, 2011) that activates a series of transcriptional factors, including the Mitochondrial transcription factor A (TFAM), which regulates transcription and replication of mtDNA (Kang et al., 2018), and Nuclear respiratory factor 1 (NFR-1) and 2 (NFR-2), which control the mitochondrial protein-encoded nuclear genes (Scarpulla, 2011).

The buffer of intracellular Ca^2+^ is mediated mainly by the cooperation between endoplasmic reticulum (ER) and mitochondria through the formation of contact sites (Krols et al., 2016) that permit the Ca^2+^ uptake from the cytosol and the exchange of the ion between the two organelles (Rizzuto and Pozzan, 2006). Ca^2+^ regulates important mitochondrial metabolic enzymes (McCormack et al., 1990). The mitochondria contain two types of Ca^2+^ channels: the Mitochondria calcium uniporter (MCU) with high selectivity for this ion and localized in the IMM (De Stefani et al., 2011) and the Voltage-dependent anion channel (VDAC) localized in the OMM that regulates the release of the Ca^2+^ from the mitochondria (Krols et al., 2016). Furthermore, VDAC cooperates with the adenine nucleotide transporter in the IMM and the cyclophin D (CypD) in the matrix on the formation of the mitochondrial permeability transition pore (mPTP) (Bernardi, 1999). An mPTP opening leads to activation of apoptosis and then cell death (Green and Kroemer, 2004). As already mentioned above, at the synaptic level, mitochondria regulate the amount of Ca^2+^ fundamental for neurotransmission and in general for the exertion of synaptic functions (Werth and Thayer, 1994; Billups and Forsythe, 2002).

Mitochondrial functions and eventually cellular homeostasis are guaranteed by a dedicated mitochondrial quality control system (mtQCS). The mtQCS comprises a multitude of different biochemical mechanisms that act at different levels, affecting individual polypeptides as well as the whole organelle. While the folding state and activities of mitochondrial proteins are controlled by endogenous chaperones and proteases (Voos, 2013), damaged mitochondria may be removed by a selective autophagy pathway, termed mitophagy (Youle and Narendra, 2011). The primary regulator of the mitophagy is a specialized signaling system consisting of the protein PTEN-induced kinase 1 (Pink1) and the ubiquitin ligase Parkin that is activated after the loss of mtΔΨ (Rüb et al., 2017). An accumulation of Pink1 at the OMM of damaged mitochondria is thought to recruit Parkin that leads to a labeling of the mitochondria for the subsequent mitophagy process. This is followed by the formation of an autophagosomal membrane engulfing the mitochondria followed by its fusion with the lysosomes where ultimately the digestion of the mitochondrial material takes place.

## Mitochondrial dysfunction in AD

In AD brain, the alteration of energetic pathways, also linked to the reduction of glucose consumption, is a well-established feature of the disease (Gibson and Shi, 2010). The glucose uptake in the brain is usually measured with the positron emission tomography (PET) tracer 18-fluorodeoxyglucose (fDG). In subjects with AD, PET studies have consistently demonstrated a low rate of glucose metabolism (between 20% and 30% lower than healthy individuals) in brain regions involved in processing memory (e.g., the hippocampus, posterior cingulate, temporal, and parietal lobes) (Kapogiannis and Mattson, 2011). Furthermore, it was proposed that the metabolic changes appeared earlier than the onset of the histopathological markers and symptoms (Gibson and Shi, 2010). Although the real cause is still unclear, the defective metabolism that characterizes AD could be easily linked to mitochondrial dysfunction.

Since its formulation in 1992 (Hardy and Higgins, 1992), the “amyloid cascade hypothesis” has dominated the AD field in the past 30 years. This hypothesis was based on two clear evidences: Aβ peptides constitute the extraneuronal senile plaques and mutation of Aβ peptides precursor, amyloid-β precursor protein (APP), leads to an early onset of AD. However, due to the fails in all Phase III clinical trials in human AD, this hypothesis has substantially lost ground and needed to be strongly revised or integrated with other hypotheses (Karran et al., 2011). In 2004, a new hypothesis was proposed to explain the onset of sporadic AD. The hypothesis, called “mitochondrial cascade hypothesis,” described that each human genetic heritage influences mitochondrial functions with a primary repercussion on the onset of AD pathology. In other words, according to this hypothesis, the mitochondrial dysfunction is the primary process to trigger all the cascade of events that lead to sporadic late-onset AD (Swerdlow and Khan, 2004; Swerdlow et al., 2014). 

Despite the fact that the validity of the mitochondrial cascade hypothesis has yet to be demonstrated in different AD models as well as human patients, the following mitochondrial functions were found severely compromised in the AD context (Hauptmann et al., 2009): mitochondrial morphology (Johnson and Blum, 1970) and number (Hirai et al., 2001), oxidative phosphorylation, mtΔΨ, Ca^2+^ buffering, ROS production (Butterfield and Halliwell, 2019), mtDNA oxidation and mutation (Wang et al., 2006), mitochondrial-ER contact sites (Area-Gomez et al., 2018), mitochondrial biogenesis, mitochondrial transport along the neuronal axon (Calkins and Reddy, 2011), and mitophagy (**Figure 1**). In a neuronal context, any of these dysfunctional processes could lead to synaptic deficits and critical consequences not only for single neurons but also for a more complex structure like the brain (Cai and Tammineni, 2017). 

In AD brains, the activities of the enzymes involved in mitochondrial energy production, such as complex IV cytochrome c oxidase (COX), pyruvate dehydrogenase complex, mitochondrial isocitrate dehydrogenase, α-ketoglutarate dehydrogenase (αKGDH), and ATP synthase complex were found decreased, while the succinate dehydrogenase (complex II) and malate dehydrogenase activities were increased (Maurer et al., 2000; Cardoso et al., 2004; Gibson and Shi, 2010; Wojsiat et al., 2015). This definitely compromises the maintenance of the mtΔΨ and eventually of the mitochondrial ATP production (Beck et al., 2016).

In line with that, the imbalance between ROS production and antioxidant power was observed in AD brains, cerebrospinal fluid (CSF), and blood (García-Blanco et al., 2017). Since the 1990s, the ROS-induced oxidative stress has received considerable attention as one of the main factors contributing to the AD pathogenesis (Mark et al., 1997). Already the mild cognitive impairment (MCI), an early stage in the AD chronology, is characterized by the significant increase of oxidative stress markers, such as lipid peroxidation and protein oxidation products, and the decrease of antioxidants in the brain and peripheral compartments (Praticò et al., 2002; Rinaldi et al., 2003; Butterfield et al., 2006).

The analysis of the samples from different AD experimental models and AD patients showed a strong link between the oxidative stress and mitochondrial dysfunction. In the transgenic mice over-expressing human APP (Tg mAPP mice), an early and progressive accumulation of Aβ peptide in synaptic mitochondria led to a mitochondrial synaptic dysfunction such as damaged mitochondrial respiratory activity, increased mPTP and oxidative stress, and impaired mitochondrial axonal transport (Du et al., 2010). Data from the 3xTg-AD mice showed that the compromised mitochondria bioenergetics together with elevated oxidative stress levels are early phenomena appearing before the development of observable Aβ plaques (Hauptmann et al., 2009; Yao et al., 2009). Oxidation of one of the mitochondrial enzymes involved in the oxidative phosphorylation, ATP synthase, was found in isolated lymphocytes from AD peripheral blood as well as in MCI and AD brains (Sultana et al., 2006; Reed et al., 2008; Tramutola et al., 2018). This may explain the compromised activity of the ATP synthase and the reduction of ATP levels in AD. Another paper showed a correlation between the reduction of the mitochondrial enzyme Aconitase (ACO2) activity and the plasma antioxidant levels in peripheral lymphocytes from MCI and AD patients proving again the strong association between the oxidative stress and the mitochondrial dysfunction in AD (Mangialasche et al., 2015). Interestingly, the new and innovative technology for AD modeling obtained with the human induced pluripotent stem cells (iPSCs) directly from AD patients demonstrated further that AD-relevant mitochondrial aberrations, including oxidative stress, have a causative role in the developments of the disease. Indeed, neurons and astrocytes from AD-iPSCs presented increased ROS production and RCC levels and enhanced susceptibility to the stressors (Ochalek et al., 2017; Oksanen et al., 2017; Birnbaum et al., 2018).

The mitochondrial dynamics such as fusion and fission processes were found unbalanced in AD, potentially leading to i) compromised distribution and morphology of mitochondria in the neurons (Hirai et al., 2001) and ii) fragmented mitochondria observed in fibroblasts and brains from AD patients (Wang et al., 2008a; Wang et al., 2009). The mitochondrial fusion and fission proteins were differentially expressed in AD hippocampus with an increase of the mitochondrial fission protein Fis1 alongside with a significant downregulation of Drp1 and fusion proteins Mfn1, Mfn2, and OPA1 (Wang et al., 2009). Similar results were found in a AD cybrids model, together with bleb like- and shorter mitochondria compared to control samples (Gan et al., 2014). Furthermore, increased phosphorylation at Ser 616 site and S-nitrosylation of Drp1, which both facilitate the mitochondrial fission (Taguchi et al., 2007; Cho et al., 2009), were higher in a AD brains compared to control (Wang et al., 2009). Beside that, the protein Drp1 was seen interacting with Aβ and phosphorylated tau in brain homogenates from AD patients (Manczak et al., 2011; Manczak and Reddy, 2012). A recent study performed in samples from AD and healthy control subjects showed the significant association between a specific polymorphism in *MFN2* gene and AD suggesting that genetic polymorphism of fusion process regulation might be involved in the AD pathogenesis (Kim et al., 2017). In addition, mfn2 protein act as a tether between mitochondria and ER membranes (de Brito and Scorrano, 2008). In this regard, mfn2 influences the Presenilin 2 (PS2), whose mutation is linked to the familial AD (FAD), in the modulation of the mitochondria-ER contact sites (Filadi et al., 2016). 

Several experimental AD models linked to APP overexpression or Aβ peptides treatments are characterized as well by mitochondrial fragmentation and abnormal mitochondrial distribution along the neurons due to an alteration of mitochondrial fusion and fission proteins levels (Wang et al., 2008b; Du et al., 2010; Zhao et al., 2010; Calkins and Reddy, 2011; Wang et al., 2017). All these results lead to two critical remarks: i) the altered balance between fusion and fission that interferes with mitochondrial transport contributes actively to the AD pathogenesis and ii) the mitochondrial dynamics impairment could be a new therapeutic target in AD.

Another key mitochondrial function, the mitochondrial biogenesis, was impaired in AD. The significant reduction of the number of mitochondria in AD human hippocampus and in cell culture models already suggests that the mitochondrial biogenesis is compromised (Hirai et al., 2001; Wang et al., 2008b). Furthermore, the level of protein regulating the mitochondrial biogenesis such as PGC-1α, NRF1 and 2, and TFAM was significantly reduced in human AD hippocampus and cellular models overexpressing APP Swedish mutation (Qin et al., 2009; Sheng et al., 2012). In the AD mouse model harboring mutant human transgenes of APP and Presenilin-1 (PS1), the mitochondrial biogenesis markers were found again declined in particular in the hippocampus region, and the use of melatonin brought beneficial effects (Song et al., 2018).

Interestingly, on one side, mitophagy was able to reverse the memory impairment, to prevent the cognitive deterioration and the Aβ peptide/tau pathology in several AD models (Fang et al., 2019). However, on the other side, mitophagy was also strongly affected in AD, leading to the accumulation of damaged mitochondria and consequently to dysfunctional neurons. One cause may be the impairment of the fusion between the autophagosome and lysosomes. This was observed in cultured cells overexpressing mutant APP, in AD mouse models, and also in neurons from AD patients’ brain (Boland et al., 2008; Lee et al., 2010; Coffey et al., 2014). In AD brains, the somatic mutations found in mtDNA are higher than in healthy brains, potentially triggering other neuropathological consequences such as the increased ROS production in neurons and the promotion of amyloidogenic processing of APP (Lin et al., 2002).

The two major and typical histopathological markers of AD, Aβ peptide and tau, harmfully accumulate in or interact non-specifically with mitochondria (Eckert et al., 2010). Aβ peptide and abnormal tau negatively affect axonal transport and consequently the transport of mitochondria along the axon from the neuronal soma to the synapses. AD mouse models, overexpressing Aβ peptides, have damaged mitochondria usually characterized by impaired axonal transport of mitochondria, a reduced mtΔΨ, and inhibited RCC with a compromised ATP production (Rui et al., 2006). The accumulation of Aβ peptides or of the precursor APP inside the mitochondria (Anandatheerthavarada et al., 2003; Hansson Petersen et al., 2008) and even the interaction of Aβ peptides with some component of the mitochondrial matrix (Lustbader et al., 2004) would be the most straightforward and rational explanations to justify the mitochondrial dysfunctions in the animal models of AD. However, mitochondria lack APP and the set of the enzymes required for Aβ peptide generation, making a mitochondria-localized production of Aβ peptides unlikely. Furthermore, a solid mechanism that explains the mitochondrial import of Aβ peptides and the direct negative effects of Aβ peptides on mitochondria is still missing, suggesting that the mitochondrial dysfunctions identified in all these AD models are indirect effects of Aβ peptides. In support of this point, a recent study showed that Aβ peptides impaired mitochondrial import of nuclear-encoded precursor proteins due to an extra mitochondrial co-aggregation process (Cenini et al., 2016).

Tauopathies including AD are also characterized by mitochondrial dysfunction. Tau influences, directly and indirectly, the mitochondrial transport along the neuronal axon and the mitochondrial functions. This leads to the reduction and impairment of mitochondria at the presynaptic terminals with obvious deleterious consequences (Dubey et al., 2008; DuBoff et al., 2012). In AD brains, phosphorylated tau was found interacting with VDAC1 leading as well to mitochondrial dysfunction (Manczak and Reddy, 2012). Hyperphosphorylation of tau negatively affects complex I activity with a decrease of ATP production, an increase of oxidative stress, dissipation of mtΔΨ, induction of the mitochondrial fission, and excessive mitochondrial fragmentation in postmortem brains from AD patients and in murine models (Manczak et al., 2011; Eckert et al., 2014). In addition, mitochondrial stress was shown to promote tau-hyperphosphorylation in a mouse model (Melov et al., 2007). These observations argue for a prominent role of tau pathology in the mitochondrial dysfunction of AD. 

The Translocase of outer membrane 40 kDa submit homolog (Tomm40) is a mitochondrial channel localized in OMM that is fundamental for the import of nuclear-encoded mitochondrial preproteins (Chacinska et al., 2009). Aβ peptides affected directly or indirectly the mitochondrial import machinery including Tomm40, and this may also contribute to the mitochondrial dysfunction observed in AD (Devi et al., 2006; Anandatheerthavarada and Devi, 2007; Cenini et al., 2016). *TOMM40* gene is contained in a tight gene cluster together with APOE gene in the chromosome 19 (Gottschalk et al., 2014; Subramanian et al., 2017). APOE is one of the most significant genetic risk factors for late-onset sporadic AD (LOAD) with the ε4/ε4 isoform linked to the highest risk (Saunders et al., 1993). It seems that also a variable-length, deoxythymidine homopolymer polymorphism in intron 6 of the *TOMM40* gene represents a genetic risk for LOAD. However, different groups showed that *TOMM40* SNPs (single-nucleotide polymorphisms) are associated with the LOAD (Martin et al., 2000; Takei et al., 2009; Kim et al., 2011; Davies et al., 2014). In a Caucasian ethnic group three variants of the *TOMM40* polymorphisms were identified, and the variant rs10524523 has received particular attention since it lowered the age of LOAD onset by 7 years in APOE3/4 carriers (Roses et al., 2010). Furthermore, this variant was associated with impaired cognition and the gray matter volume in the brain area susceptible to AD (Johnson et al., 2011). Different groups also demonstrated the strong influence of *TOMM40* “523” variant on *TOMM40* and *APOE* genes transcription (Linnertz et al., 2014; Payton et al., 2016).

The integration of all these facts into a significant biological context like neuronal cells in AD, suggests that the accumulation of dysfunctional mitochondria at the synapses and the lack of their replacement would contribute substantially to the neurons degeneration and consequently to the worsening of the AD condition.

## Mitochondrial therapies in AD

AD is still without a cure and also essentially lacks a rational understanding of the primary event triggering the disease. Nevertheless, an improved comprehension of this deleterious disorder and the development of effective treatments are essential not only to heal the disease but also eventually to prevent or postpone the onset of the symptoms in the patients. 

The traditional cures used nowadays to treat the AD patients are so far the cholinesterase inhibitors (**donepezil**, **rivastigmine**, and **galantamine**) and **memantine** that block the N-methyl-D-aspartate (NMDA) receptor and the excess of glutamate activity. NMDA receptors and acetylcholin (Ach) are fundamental in memory and learning processes and their concentration and function are compromised in AD (Francis, 2005). However, these treatments improve the cognitive and memory functions, without really slowing down the progression of the disease.

As described above, mitochondrial dysfunctions and a compromised energetic metabolism are two prominent aspects of AD pathology. Therefore, mitochondria should be seriously considered as pharmacological targets. In the course of history, nevertheless, different compounds affecting mitochondria were already tested in AD without a successful outcome. However, as the idea of AD as a multifactorial disease gained more ground in the last years, a reconsideration of mitochondria as a valid therapeutic target together with other medications is strongly recommended.

**Figure 2:**
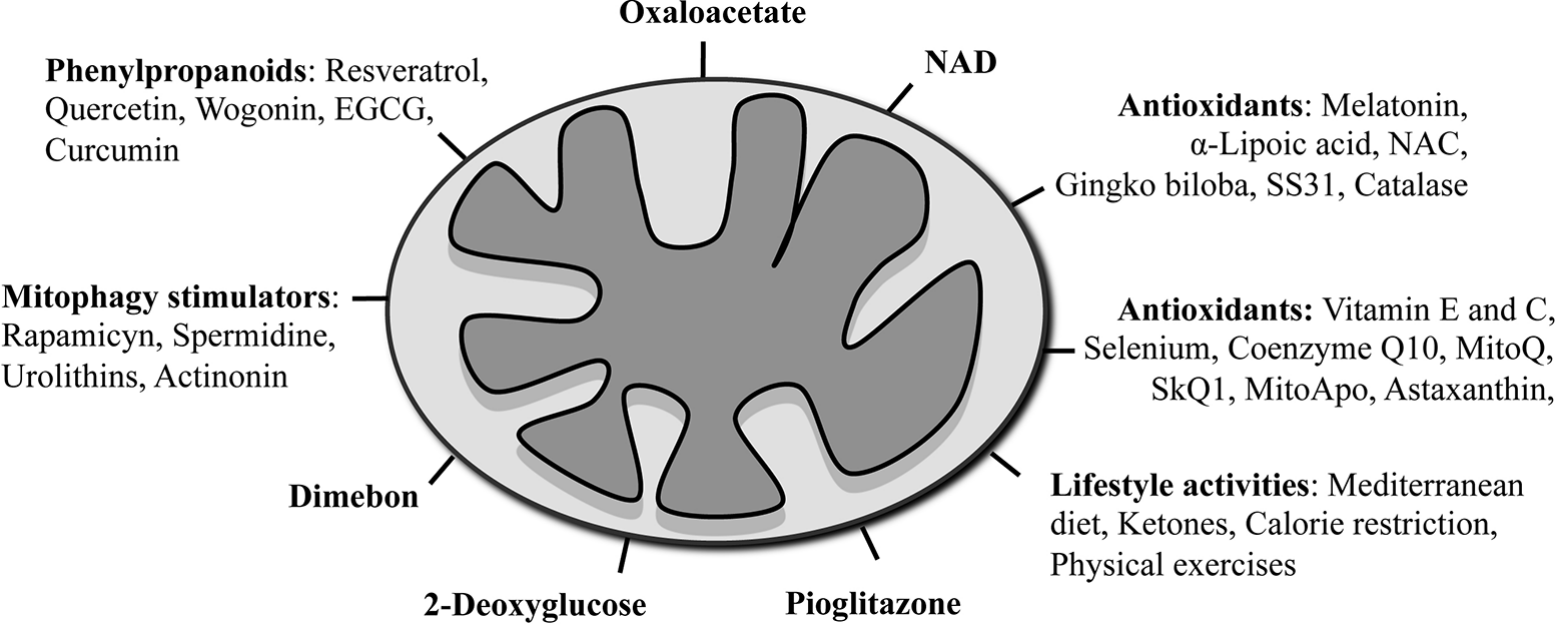
Schematic summary of mitochondrial-targeted therapies used in AD models and clinical trials.

Mitochondria could be targeted through two ways: i) by pharmacologic approaches acting on mitochondria directly or ii) by action on the lifestyle that indirectly hits this organelle (**Figure 2**). In the following section, we describe the most popular mitochondrial treatments that have been used until today on AD patients, and in **Table 1**, we summarize specifically the beneficial effects of these compounds on mitochondria in different experimental AD models. The table is also a proof that these treatments are able to act effectively and positively on mitochondria, and therefore a revision and improvement of their use in AD would be worthy.

**Table 1:** List of compounds and lifestyle activities effects on mitochondria in experimental models for AD.

Treatment	Effect on mitochondria	Experimental AD models	References
**Antioxidants**			
*Vitamin E*	Increase mtΔΨ and ATP ROS scavenger Reduction of lipid peroxidation	*In vitro* glutamate-injured astrocytes *In vivo* aged old mice	(Selvaraju et al., 2014; Schloesser et al., 2015)
*Selenium*	Inhibition of ROS production and oxidative damage Reduction of mitochondrial membrane depolarization	*In vitro* Aβ*_42_*-*CFP*-overexpressed HEK293 cell line In vivo scopolamine-treated aged rats	(Chen et al., 2013; Balaban et al., 2017)
*Vitamin C*	Maintenance of mitochondrial integrity through reduction of oxidative damage Reduction of mitochondrial membrane depolarization and mitochondria-mediated apoptosis	*In vitro* Aβ_1-42_ peptide-treated human cortical neurons *In vivo* 5XFAD Tg mice *In vivo* APP/PSEN1 mice	(Medina et al., 2002; Kook et al., 2014; Dixit et al., 2017)
*Coenzyme Q10*	Attenuation of decreased oxidative phosphorylation efficiency and of increased H_2_O_2_ production Reduction of mitochondrial accumulation of Aβ peptide Prevention of Aβ peptide-induced mPTP opening Protection against dissipation of mtΔΨ Beneficial effect of mitochondrial ETC	Isolated mitochondria from Aβ_1-40_ peptide-treated diabetic Goto–Kakizaki aged rats *In vitro* Aβ_25-35_ peptide-treated HUVEC cell line *In vitro* Aβ_1-42_ peptide-treated M17 cell line *In vivo* TgP301S mice *In vivo* Tg19959 mice	(Moreira et al., 2005; Dumont et al., 2011; Elipenahli et al., 2012; Sadli et al., 2013; Durán-Prado et al., 2014)
*Mitoquinone (MitoQ)*	Prevention of increased ROS production, loss of mtΔΨ, decreased GSH/GSSG ratio, increased MDA and 3-NT Regulation of mitochondrial fusion, fission, and matrix genes Protection of mitochondrial structure Amelioration of ATP production, COX activity, and depletion of the cardiolipin	*In vitro* Aβ_22-35_ peptide-treated mouse cortical neurons and N2a cell line *In vivo* 3xTg-AD and Tg2576 mice *In vivo* human Aβ-overexpressed *C. elegans*	(Manczak et al., 2010; McManus et al., 2011; Ng et al., 2014)
*SkQ1*	Preservation of mitochondrial structure Improvement of mitochondrial biogenesis Increase of COX activity Inhibition of ROS production Reduction of mtDNA deletion	*In vivo* OXYS rats	(Loshchenova et al., 2015; Stefanova et al., 2016; Kolosova et al., 2017)
*MitoApo or apocynin*	Protection against oxidative stress-induced cell death Reduction of superoxide production	*In vitro* 6-OHDA-treated LUHMES cell line	(Brenza et al., 2017)
*Astaxanthin*	Prevention of mitochondrial H_2_O_2_ production	*In vitro* Aβ_1-42_ oligomers-treated mouse hippocampal neurons	(Lobos et al., 2016)
*Melatonin*	Restoration of: respiration rate, RCC proteins expression, mtΔΨ, ROS production, ATP levels Prevention of decreased mitochondrial volume Improvement of mitochondrial biogenesis factors expression and mtDNA/nuDNA ratio Amelioration of mitochondrial membrane fluidity and mitochondrial structure Stabilization of cardiolipin and mPTP Decrease of mitochondrial Ca^2+^ levels	Isolated mitochondria from APPswe and APP/PSEN1 mice *In vitro* APPswe-overexpressed HEK293 cell line *In vitro* Aβ_22-35_ peptide-treated cultured rat hippocampal neurons *In vitro* Aβ peptide-treated NARP cybrids cell line *In vivo* OXYS rats *In vivo* injection of Aβ_1-42_ peptide in rats hippocampus *In vivo* APP/PSEN1 mice	(Dong et al., 2010; Dragicevic et al., 2011a; Dragicevic et al., 2012; Peng et al., 2012; Rosales-Corral et al., 2012b; Gerenu et al., 2015; Rudnitskaya et al., 2015; Wang et al., 2019)
*α-Lipoic acid (LA)*	Decrease of oxidative stress and apoptotic markers Preservation of COX assembly Elevation of ATP levels, Krebs cycle dehydrogenase, complex I, and COX activities	*In vitro* AD fibroblast *In vivo* aged rats *In vitro* Aβ_1-42_ peptide-treated differentiated SH-SY5Y cell line *In vivo* ApoE4 Tg mice	(Moreira et al., 2007; Ajith et al., 2014; Marinelli et al., 2017)
*N-Acetyl-cysteine (NAC)*	Decrease oxidative stress and apoptotic markers Preservation of COX assembly	*In vitro* AD fibroblast	(Moreira et al., 2007)
*Ginkgo biloba*	Stabilization of mtΔΨ and ATP production Reduction of ROS/RNS production Increase of mitochondrial APE1 levels Enhancement of complex I, III, COX activities Improvement of oxygen consumption Up-regulation of mitochondrial DNA Block of mitochondria-mediated apoptosis	*In vitro* APPmutant-overexpressed and Aβ peptide-treated PC12 cell line *In vitro* Aβ_25-35_ peptide-treated IMR-32 and SH-SY5Y cell line *In vitro* APP-overexpressed SH-SY5Y cell line *In vivo* Aβ_25-35_ peptide-injected rats	(Eckert et al., 2003; Eckert et al., 2005; Rhein et al., 2010; Tian et al., 2013; Kaur et al., 2015)
*Szeto-Schiller tetrapeptides 31 (SS31)*	Increase of mitochondrial biogenesis and dynamics proteins level Rescue of mitochondrial anterograde transport ROS scavenger and reduction of H_2_O_2_ and lipid peroxidation levels Prevention of mPTP, mitochondrial swelling, and mitochondria-mediated apoptosis Protection of mitochondrial structure Increase of ATP production and supply at nerve terminals Increase of COX activity, and mtΔΨ Increase of mtDNA copy number and mitochondrial network	*In vitro* primary neurons from Tg2576 mice *In vitro* Aβ_22-35_ peptide-treated or APPswe and APPInd-overexpressed N2a cell line *In vivo* Tg2576 mice	(Manczak et al., 2010; Calkins et al., 2011; Reddy et al., 2017, Reddy et al., 2018)
*Catalase*	Reduction of abnormal APP process, oligomeric Aβ peptides, and BACE1 activity and levels, and oxidative damage Increase of protective soluble APPα and CTF83 fragments	*In vivo* MCAT/APP mice	(Mao et al., 2012)
**Phenylpropanoids**			
*Resveratrol*	Attenuation of ROS accumulation, mtΔΨ, and mitochondria-mediated apoptosis Increase of COX levels Stimulation of mitophagy/autophagy	*In vitro* Aβ peptide-treated PC12 cell line *In vivo* APP/PSEN1 mice	(Jang and Surh, 2003; Porquet et al., 2014; Deng and Mi, 2016; Wang et al., 2018)
*Quercetin*	Restoration of mtΔΨ, ROS production, and ATP levels, and the normal mitochondrial morphology Increase MnSOD activity Prevention of mitochondria-mediated apoptosis	*In vivo* APP/PSEN1 mice *In vitro* Aβ peptide-treated rat hippocampal neurons *In vitro* OA-treated HT22 hippocampal neurons *In vivo* aluminum-treated rats	(Wang et al., 2014; Jiang et al., 2016; Sharma et al., 2016; Godoy et al., 2017)
*Wogonin*	Rescue the mtΔΨ loss Attenuation of mitochondria-mediated apoptosis	*In vitro* Tet-On Aβ42-GFP-overexpressed SH-SY5Y cell line *In vivo* 3xTg-AD mice	(Huang et al., 2017)
*Epigallocatechin-3-gallate (EGCG)*	Attenuation of ROS accumulation Increase of MnSOD level Restoration of altered mtΔΨ_t_, ATP levels, and mitochondria respiratory rates	Isolated mitochondria from hippocampus, cortex, and striatum of APP/PSEN1 mice *In vitro* APP695-overexpressed N2a cell line *In vitro* APPmut-overexpressed neuroblastoma cell line *In vivo* streptozotocin-infused Wistar rats	(Dragicevic et al., 2011b; Biasibetti et al., 2013; Zhang et al., 2017)
*Curcumin*	Increase of ATP levels and COX activity Positive effect on mtΔΨ and respiratory control ratio Reduction of ROS production and mitochondria-mediated apoptosis Restoration of complex I, II, COX levels and activities	*In vitro* Aβ_22-35_ peptide-treated SH-SY5Y cell line *In vitro* glutamate-treated PC12 cell line *In vivo* APP751SL mice *In vivo* APP/PSEN1 mice *In vivo* aluminum-treated rats	(Sood et al., 2011; Chang et al., 2014; Hagl et al., 2014; Gerenu et al., 2015; Reddy et al., 2016)
**Action of the life style**			
*Calories restriction*	Decrease of F_0_F_1_-ATPase activity	*In vivo* P301L mice	(Delic et al., 2015)
*Oleuropein aglycone (OLE)*	Stimulation of mitophagy/autophagy	*In vivo* TgCRND8 mice	(Grossi et al., 2013; Pantano et al., 2017)
*Hydroxytyrosol (HT)*	Reduction of mitochondrial carbonyl protein ROS scavenger Enhancement of MnSOD level	*In vivo* APP/PSEN1 mice *In vitro* copper-treated SH-SY5Y cell line	(Peng et al., 2016; Omar et al., 2017)
*Ketones*	Increase of TCA cycle intermediates and ATP hydrolysis Reduction of mitochondrial redox potential (free mitochondrial [NAD^+^]/[NADH] ratio oxidation)	*In vivo* 3xTg-AD mice	(Pawlosky et al., 2017)
*Physical exercise (PE)*	Increase of mitochondrial mass, mtΔΨ, complexes I, COX, αKGDH, and ATP synthase activities Reduction of ROS production and mtDNA oxidative damage Restoration of mitochondrial antioxidant enzymes and OGG1 activities Suppression of OGG1 and MnSOD acetylation Modulation of mitochondrial dynamics proteins (Mfn1 and Drp1)	Isolated mitochondria from APP/PSEN1 mice *In vivo* swimming-trained pregnant rats *In vivo* 3xTg-AD mice	(Bo et al., 2014; Klein et al., 2019)
*2-deoxyglucose*	Increase of αKGDH level Reduction of mitochondrial APP and Aβ oligomer level, mitochondrial stress response proteins levels, mtΔΨ	*In vivo* 3xTg-AD mice *In vivo* Aβ peptides-treated adult rats	(Guo and Mattson, 2000; Yao et al., 2011)
*Rapamycin*	Prevention of decrease of mtΔΨ Stimulation of mitophagy/autophagy	*In vitro* Aβ_1-42_ peptide-treated PC12 cell line	(Xue et al., 2016)
*Spermidine, Urolithin A, Actinonin*	Stimulation of mitophagy/autophagy	*In vivo* Aβ and tau *Caenorhabditis elegans* models *In vivo* APP/PSEN1 mice	(Fang et al., 2019)
**Other mitochondrial-based therapy**			
*Nicotinamide adenine dinucleotide (NAD)*	Prevention of OCR deficits Promotion of PGC-1α level Restoration of NAD^+^ and ATP level Changes of mitochondrial dynamics fusion–fission Block of ROS accumulation Stimulation of mitophagy/autophagy	*In vitro* APP/PSEN1-overexpressed hippocampal neuroblastoma *In vitro* NMN-treated organotypic hippocampal slice cultures (OHCs) *In vivo* APP/PSEN1 mice *In vivo* Aβ oligomer-infused rats *In vivo* Tg2576 mice *In vivo* Aβ and tau *Caenorhabditis elegans* models	(Long et al., 2015; Wang et al., 2016; Fang et al., 2019)
*Pioglitazone*	Restoration of mitochondrial energy metabolism and activity	Isolated mitochondria from APP/PSEN1 mice *In vitro* APP695-overexpressed CHO cell line	(Chang et al., 2015, Chang et al., 2019)
*Dimebon (Latrepirdine)*	Increase and maintenance of succinate dehydrogenase and RCC activities, mtΔΨ, ATP levels, TIM and TOM proteins levels, mitochondrial dynamics and morphology Attenuation of Ca^2+^ induced mitochondrial swelling Restoration of impaired autophagy/mitophagy and mPTP proteins levels	Isolated mitochondria from rat *In vitro* mouse cortical neurons and SH-SY5Y *In vitro* APPswe-overexpressed HEK293 cell line *In vitro* glutamate-treated CGNs	(Zhang et al., 2010; Naga and Geddes, 2011; Eckert et al., 2012; Weisová et al., 2013)

AD, Alzheimer’s disease; ETC, electron transport chain; RCC, respiratory chain complexes, mtΔΨ: mitochondrial membrane potential; OCR, oxygen consumption rates; ATP, adenosine triphosphate; mPTP, mitochondrial permeability transition pore; mtDNA, mitochondrial deoxyribonucleic acid; nuDNA, nuclear deoxyribonucleic acid; APE1, apurinic/apyrimidinic endonuclease 1; MnSOD, manganese superoxide dismutase; OGG1, oxoguanine DNA glycosylase-1; αKGDH, α-ketoglutarate dehydrogenase; COX, cytochrome c oxidase or complex IV; TIM, translocase inner membrane; TOM, translocase outer membrane; Mfn1, mitofusin-1; Drp1, dynamin-1-like protein; PGC-1α, peroxisome-proliferator-activated receptor γ coactivator-1α; NAD, nicotinamide adenine dinucleotide; NADH, reduced nicotinamide adenine dinucleotide; ROS, reactive oxygen species; RNS, reactive nitrogen species; GSH, glutathione; GSSG, oxidized glutathione; 3-NT, 3-nitrotyrosine; MDA, malondiaaldehyde; SelM, selenoprotein M; 6-OHDA, 6-hydroxydopamine; OA, okadaic acid; H_2_O_2_, hydrogen peroxide; NMN, nicotinamide mononucleotide; Aβ, β-amyloid peptide; AβPP, β-amyloid precursor protein; PS1, presenilin 1; BACE1, β-secretase-1; HEK293, human embryonic kidney 293 cell lines; HUVEC, human umbilical vein endothelial cell line; M17, human neuroblastoma cell line; N2a, mouse neuroblastoma cell line; LUHMES, Lund human mesencephalic cell line; SH-SY5Y, human neuroblastoma cell lines; IMR-32, human neuroblastoma cell lines; PC12, pheochromocytoma of rat adrenal medulla-derived cell lines; OHCs, organotypic hippocampal slice cultures; NARP, cybrid cell lines bearing mtDNA mutation T8993G; CGN, cerebellar granule neurons; 5xFAD, mice expressing human APP and PSEN1 genes with a total of five AD-linked mutations, the Swedish, Florida, and London mutations in APP, and the M146L and L286V mutations in PSEN1; APP/PSEN1, mice contain human APP gene bearing the Swedish mutation and PS1 gene containing L166P mutation; TgP301S, mice expressing mutant human microtubule-associated protein tau (MAPT); Tg19959, mice expressing human APP gene bearing the Swedish mutation and Indiana mutation; TgCRND8, mice expressing human APP695 gene with the Swedish mutation and Indiana mutation; 3xTg-AD, mice contain three mutations (Swedish, MAPT, PS1) associated with familial AD; Tg2676 mice, mice expressing mutant human form of APP (isoform 695) with Swedish mutation; APP751SL, mice expressing the human APP bearing both Swedish and the London mutation; ApoE4 Tg mice, mice expressing human apolipoprotein E (APOE) gene; OXYS rats, senescence-accelerated rats; MCAT, mitochondria-targeted catalase; C. elegans, Caenorhabditis elegans.

More information about the ongoing clinical trials concerning mitochondria in AD are summarized in Wilkins et al. and in Perez Ortiz et al. (Perez Ortiz and Swerdlow, 2019; Wilkins and Morris, 2017), and they can also be found in www.clinicaltrials.gov. 

### Antioxidants

Since the increased oxidative stress accompanied by the reduction of the antioxidant power was measured in the brain, CSF, and blood from AD patients, treatments with antioxidant compounds were tested to counteract this oxidative unbalance and slow down the progression of the AD symptoms. 

Typical antioxidants were the **vitamins**, **E** and **C**, but their effects in the context of AD remain questionable. For example, in two studies with vitamin E, some markers of lipid peroxidation were found decreased in AD patients’ CSF, with no consistent effect on or even a deterioration of cognitive functions (Arlt et al., 2012; Galasko et al., 2012). Vitamin E was also administered in combination with **selenium**. However, high levels of selenium were found toxic with a pro-oxidant effect, glial activation, and neuronal death (Vinceti et al., 2014). There is an important study called **PREADViSE** that was performed to see the long-term effect of anti-oxidant supplements (Vitamin E, selenium, Vitamin E + selenium or placebo) on dementia incidence among asymptomatic men. However, the supplement did not prevent dementia occurrence (Kryscio et al., 2017).

Targeting directly the mitochondria with antioxidant compounds was always one of the most considered therapeutic strategies in AD. In this regard, an antioxidant directed to mitochondria that has been tried was the **coenzyme Q10** (**CoQ10**). CoQ10 has a quinone structure and is a component of the mitochondrial RCC. In a rat model for AD, CoQ10 prevented the cognitive decline (Dehghani Dolatabadi et al., 2012). Still, due to a low bioavailability in the brain (Kwong et al., 2002), CoQ10 has never been successful in humans. To overcome this issue, the **mitoquinone mesylate** (**MitoQ**) was optimized. MitoQ is an antioxidant compound made of ubiquinone conjugate with triphenylphosphonium (TPP). The TPP is necessary to target the molecule to the mitochondria because it helps to cross the lipid bilayers accumulating on the negative site of mitochondrial membranes (Kelso et al., 2001; Smith et al., 2003). MitoQ behaved as ROS scavenger and was tested in different AD model systems (see **Table 1**). Here, MitoQ shown to prevent oxidative damage, to protect RCC activity, to reduce Aβ peptide levels, synaptic loss, and astrogliosis, and to improve cognitive functions (McManus et al., 2011; Ng et al., 2014). As reported in the review from Ortiz (Perez Ortiz and Swerdlow, 2019), at the moment, MitoQ is tested in a small clinical trial to check its effect on cerebrovascular blood flow in AD. Similarly to MitoQ, other antioxidant compounds (**SkQ1**, **MitoApo**, **astaxanthin**) affect positively the mitochondrial functions (see **Table 1**) and could be potentially used to treat AD (Lobos et al., 2016; Stefanova et al., 2016; Brenza et al., 2017). 

Another group of antioxidant molecules such as **melatonin**, **α-lipoic acid (LA)**, **N-Acetyl-cysteine (NAC)**, and ***Ginkgo biloba*** were tested *in vivo* and *in vitro* and showed protective effects on Aβ peptide accumulation and mitochondrial toxicity as well as on cognitive functions (Dong et al., 2010; Rosales-Corral et al., 2012a). **Melatonin** is a neurohormone produced by the pineal gland with neuroprotective functions in AD pathogenesis (Shukla et al., 2017). Melatonin is a ROS scavanger and showed some anti-amyloidogenic properties (Dong et al., 2010; Rosales-Corral et al., 2012a). At mitochondrial level, melatonin prevented the ROS production, the cardiolipin oxidation, and the mPTP opening, restored the Ca^2+^ balance, and reduced the caspase-3 and -9 levels (Feng and Zhang, 2004; Jou et al., 2004; Petrosillo et al., 2009; Espino et al., 2010). Treatments with **α-lipoic acid**, a cofactor for many RCC enzymes, exhibited a positive effect on cognitive functions in clinical trials on AD patients and in murine models of aging and AD, α-lipoic acid affected also the formation and the stabilization of Aβ peptide fibril as well as the protection against the Aβ peptide toxicity in cultured hippocampal neurons (Liu et al., 2002; Lovell et al., 2003; Ono et al., 2006; Hager et al., 2007; Quinn et al., 2007; Sancheti et al., 2013). **N-Acetyl-cysteine** (**NAC**) is the precursor of the endogenous antioxidant glutathione (GSH), a key molecule for the maintenance of mitochondrial functions (Traber et al., 1992). *In vitro* and *in vivo*, NAC had beneficial effects on Aβ peptide and phosphorylated tau levels with improvement of cognitive functions, protection against memory decline, and reduction of oxidative stress markers (see also **Table 1**) (Studer et al., 2001; Fu et al., 2006; Huang et al., 2010; Costa et al., 2016). In two clinical trials, subjects with MCI, AD, or early memory loss were treated for a long time with a nutraceutical formulation that also included NAC. Improvement of cognitive and behavioral functions was observed (Remington et al., 2015; Remington et al., 2016). ***G. biloba*** is a natural antioxidant already used in the Chinese traditional medicine. **Table 1** shows all the effects of *G. biloba* on mitochondrial functions. Two clinical trials were performed to test the effect of *G. biloba* in the prevention against memory and cognitive decline in older adults and AD subjects. Unfortunately, no positive effects were observed in these tests (Snitz et al., 2009; Vellas et al., 2012).

The **Szeto-Schiller** (**SS**) **tetrapeptides** are a group of small peptides that due to their structure act as antioxidants and can reach the mitochondrial matrix and the IMM (Szeto, 2006). In one of AD murine models, the **SS31** reduced Aβ peptide production, mitochondrial dysfunction, and enhanced mitochondrial biogenesis and synaptic activity (Calkins et al., 2011; Reddy et al., 2017). Recently, a combination of SS31 and the mitochondrial division inhibitor 1 (Mdivi1) was tested in cultured AD cells with positive effects, suggesting that a combined treatment of mitochondria-targeted antioxidants could have higher effectiveness (Reddy et al., 2018).

An interesting preclinical study proposed to target the antioxidant enzyme **catalase** to the mitochondria. Catalase catalyzes the decomposition of hydrogen peroxide (H_2_O_2_) in water (H_2_O) and oxygen (O_2_) and is typically localized in the peroxisome. A double transgenic mouse with mitochondria-targeted catalase (MCAT) and APP was created, and the protective effects against abnormal APP processing, Aβ peptide pathology, and lifespan extension were tested. Mitochondrial catalase showed beneficial outcomes in this highly artificial model. Although most of the antioxidant clinical trials were not entirely successful, this study proved that a direct target of an antioxidant to the mitochondria might still have a chance as a therapeutic approach in AD (Mao et al., 2012).

Despite the oxidative stress unbalance is an evident hallmark in AD and some mitochondrial-targeted antioxidant strategies showed promising effect on cognitive functions, none entered so far in the market as a valid AD treatment. There are different reasons to justify the failures (summarized in Persson et al. paper; Persson et al., 2014). The antioxidants at certain concentrations and conditions could behave as pro-oxidants and therefore they are more harmful than useful. The antioxidant administration during the clinical trials was probably started too late during the development of the disease suggesting that an early intervention could be more effective. Last, the antioxidant bioavailability in the brain could be low due to the difficulty of these molecules to cross the blood–brain barrier (BBB) requiring a rational modification of their structure to overpass this issue. 

### Phenylpropanoids

The **phenylpropanoids** are natural compounds that exert many physiological functions crucial for the survival of plants. In this heterogeneous group of substances, many subclasses have been identified such as stilbenoids, flavonoids, curcuminoids, phenolate esters, and lignans. These compounds showed an effect against the Aβ peptide and tau pathologies, on the activation of the inflammation response, on the oxidative stress, and also on the mitochondrial dysfunction (Kolaj et al., 2018). Between others, **resveratrol**, **quercetin**, **wogonin**, **epigallocatechin-3-gallate** (**EGCG**), and **curcumin** were already tested and showed to promote mitochondrial biogenesis, to impede apoptotic pathways through inhibition of DNA fragmentation, ROS formation, and caspase-3 activation, and to reduce perturbation of mtΔΨ and ATP levels (see also **Table 1** for the effects of phenylpropanoids on mitochondria in AD models) (Lagouge et al., 2006; Davis et al., 2009; Im et al., 2012; Valenti et al., 2013; Reddy et al., 2016). Furthermore, these compounds were able to restore the mitochondrial functions in a transgenic mouse model of AD (Dragicevic et al., 2011b). In particular in an *in vitro* study, **EGCG**, a major flavonoid component of the green tea, accumulated in mitochondria and exerted a strong influence on the mitochondrial functions proposing it as pharmacological treatment in AD (Schroeder et al., 2009; Dragicevic et al., 2011b). However, phenylpropanoids have a dual effect on mitochondrial function, depending on the concentration. For example, EGCG could increase apoptosis in cultured neurons at specific concentrations, while quercetin protected cultured hippocampal cells against Aβ peptide-induced apoptosis only in low concentrations (Chung et al., 2007; Ansari et al., 2009). **Curcumin** is an antioxidant compound with massive potential for the prevention and treatment of AD. It showed beneficial effects on Tg2576 AD model mice, such as reduction of the brain oxidative stress and the neuroinflammation, but no effect in AD patients, probably due to a low bioavailability (Lim et al., 2001; Baum et al., 2008; Ringman et al., 2012). New strategies have been implemented to overpass this limitation and improve the curcumin pharmacokinetics, such as the nanotechnology-based delivery system, new pharmaceutical formulations, and the change in the way of administration (Reddy et al., 2014; Serafini et al., 2017). 

Like the antioxidant, the use of the phenylpropanoids in AD treatment needs to be considered with caution and none of them has become a real therapy yet. The new AD clinical trials based on this group of molecules definitely require a broad design, a substantial revision, and a careful implementation.

### Action on the Lifestyle

#### Calories Restriction, Diet, Exercises

Lifestyle activities, in particular **exercise** and **diet**, have been known to act at the mitochondrial level and should therefore be considered as possible interventions to treat AD. **Table 1** reports the effects of the compounds and activities strictly related to the lifestyle on mitochondria from AD models. 

A **Mediterranean diet** has been correlated to the reduction of the incidence of AD (Scarmeas et al., 2006; Karstens et al., 2019). The Mediterranean diet is mainly composed of fruits, vegetables, and omega-3 fatty acids, which are enriched in olive oil. It was observed that, for example, **polyphenol-rich extra-virgin oil** reduced mitochondria-generated oxidative stress and insulin resistance in high-fat diet fed rats (Lama et al., 2017). Another polyphenol component of olive oil called **oleuropein aglycone** (**OLE**) promoted autophagy, decreased aggregated proteins levels, and reduced the cognitive impairment in AD patients’ brain (Grossi et al., 2013; Cordero et al., 2018). **Hydroxytyrosol** (**HT**), another bioactive compound of olive oil, ameliorated mitochondrial dysfunction in an animal model of AD (Peng et al., 2016). On the other side, higher consumption of **fructose** affected negatively the mitochondrial function in hippocampus from adult rats, suggesting that fructose consumption should be actively avoided (Cigliano et al., 2018). **Ketones** are another source of energy for the brain when there is a limited amount of available glucose (Owen et al., 1967). The ketone ester diet in a model of AD (3xTgAD) had positive effects also on mitochondrial functions (Pawlosky et al., 2017). The therapeutic ketosis was suggested to reduce the AD brain pathology including the accumulation of Aβ plaques and NFT (Kashiwaya et al., 2013). Of course, the results obtained in AD murine models have to be proven in humans through clinical trials (Puchowicz and Seyfried, 2017). In this regard, there are experiments going on at the University of Kansas about the effect of a ketogenic diet (KD) on participants with AD, but no definitive results are available yet (Taylor et al., 2018; Taylor et al., 2019).

An extreme form of diet is represented by **calorie restriction** (**CR**). CR is a strong limitation on calorie intake without facing a lack of nutrients. It is well known that CR is an excellent way to extend lifespan, to increase insulin sensitivity, and to prevent age-related diseases (Mattison et al., 2017). At the mitochondrial level, CR showed positive effects by affecting mitochondrial biogenesis through the induction of NO synthetase (eNOS) (Nisoli et al., 2005). Newly synthesized mitochondria led to an increase of mitophagy, reduction of ROS, increased ATP levels, and overall improvement of the mitochondrial quality and cell bioenergetics (López-Lluch et al., 2006). Furthermore, CR affected the mtDNA content as well as the amount of TFAM-bound mtDNA in rats (Picca et al., 2013). There are ongoing clinical studies around the world concerning the effect of CR and dietary intervention on MCI (Wilkins and Morris, 2017).

**Physical exercise** (**PE**) has been demonstrated to generally benefit the health of the body and mind, affecting properties such as brain plasticity and cognitive function. Hence, it could be a good prevention for age-related diseases (Hernández et al., 2015; Paillard et al., 2015). It is well known that PE targets mitochondria and improved mitochondrial function (see **Table 1** to check the effects of PE on mitochondria in AD models). A study showed that PE increased mtDNA repair, ameliorated mitochondria respiratory function through the increase of RCC activity, attenuated ROS generation capacity together with a reduction of Aβ_1-42_ peptide levels, and correlated with an amelioration of cognitive function in the hippocampus from the APP/PS1 transgenic mouse model of AD (Bo et al., 2014). However, data obtained in another AD mouse model (3xTg-AD) demonstrated that short-term exercise did not augment the critical gene expression of mitochondrial biogenesis, even if the glucose metabolism was overall improved (Do et al., 2018). Maternal exercise during pregnancy resulted in a positive effect on mitochondrial function concerning the onset of AD. In this study, a protective effect against Aβ oligomer-induced neurotoxicity in the adult offspring brain rats was shown (Klein et al., 2019). Clinical trials with PE were performed in older adults with healthy as well as impaired cognitive function. Aβ_1-42_ concentration in plasma and CSF was modified. In the brain, improvements of cognitive and executive functions, and even a change of hippocampal volume and memory, were observed, together with a reduced brain atrophy (Baker et al., 2010; Erickson et al., 2011; Vidoni et al., 2015; Yokoyama et al., 2015). Of course, in these human studies, neither a direct effect of PE on mitochondria nor the molecular mechanisms of PE benefits have been proved. However, all the studies performed in animal models positively supported the hypothesis that PE may have a beneficial effect on mitochondrial functions and glucose metabolism also in humans.

Diet, CR, and PE can also be combined to improve the quality of human aging and to prevent neurodegenerative disease (Rege et al., 2017). These approaches were shown to affect mitophagy, the cellular removal mechanism for damaged mitochondria, indicating the mitophagy as a new and promising therapeutic target to prevent the progression of the diseases. Experimental evidences from rodent studies showed that fasting and exercises could have a beneficial effect not only on mitophagy but also on mitochondrial biogenesis, reduction of oxidative stress, and overall neuronal plasticity (Alirezaei et al., 2010). Other strategies to boost mitophagy in order to delay AD are the use of compounds like **2-deoxyglucose**, which protects neurons and enhances mitochondrial functions (**Table 1**) (Duan and Mattson, 1999; Yao et al., 2011). Additional molecules that promote autophagy/mitophagy are **rapamycin**, **spermidine**, **urolithins**, and the antibiotic **actinonin** (Spilman et al., 2010; Morselli et al., 2011; Ryu et al., 2016; Fang et al., 2019). The mTOR inhibitor rapamycin was already demonstrated to have beneficial effects on a mouse AD model (Spilman et al., 2010). Testing these molecules in clinical AD might be worth it.

### Other Mitochondria-Based AD Therapy

#### Oxaloacetate

Treatment with **oxaloacetate** (**OOA**), an intermediate of the Krebs cycle and gluconeogenesis, has been proposed as a new therapeutic approach for AD, and it was already tested in some AD subjects (Swerdlow et al., 2016). Studies involving OOA performed in mice showed positive effects on glycolysis, respiratory fluxes, mtDNA and mtDNA-encoded proteins, activation of mitochondrial biogenesis, hippocampal neurogenesis activity, neuroinflammation, and change in brain insulin signaling (Wilkins et al., 2014). Despite there are no studies about the direct efficacy of OOA treatment on mitochondria in AD models, clinical trials with OOA in AD are ongoing. 

#### NAD

Nicotinamide adenine dinucleotide (NAD) is an intermediate common to several mitochondrial metabolic pathways such as glycolysis, TCA cycle, and oxidative phosphorylation. Studies on *in vitro* and *in vivo* AD models proved that NAD treatments acted directly on mitochondrial functions and were beneficial (**Table 1**). In the past, the effect of a stabilized oral NAD formulation on cognitive functions in AD patients was also tested. The rationale behind this testing was based on the enhancement of the cellular bioenergetic to improve brain performance in the fight against neurodegenerative diseases. Interestingly, after 6 months of treatment, the subjects with probable AD showed no cognitive deterioration suggesting that NAD could be an excellent method to prevent the AD progression (Demarin et al., 2004). However, further studies are needed to prove NAD as an effective treatment to slow down AD.

#### Pioglitazone

The **pioglitazone** is a peroxisome proliferator-activated receptor gamma (PPARγ) agonist. PPARγ is a ligand-activated nuclear transcription factor that has a role in regional transcriptional regulation of chr19q13.32 (Subramanian et al., 2017). This region contains the *TOMM40-APOE-APOC1* genes and, as already mentioned, *TOMM40* and *APOE4* genes are risk factors for the LOAD development. Pioglitazone was able to decrease the transcription of *TOMM40*, *APOE*, and *APOC1* genes making this molecule an interesting candidate in the AD therapy (Subramanian et al., 2017). In CHO cell line overexpressing APP695 isoform, pioglitazone lowered the Aβ_1-42_ level and restored the mitochondrial activity (Chang et al., 2015). These results were then confirmed *in vivo* in APP/PSEN1 mice (**Table 1**) (Chang et al., 2019). 

Pioglitazone is usually used to treat diabetes mellitus type 2. Some years ago, the pharmaceutical company Takeda used this compound in a large and global Alzheimer’s prevention study called TOMMORROW to slow down the progression from MCI to AD. The people involved were selected based on their *APOE* and *TOMM40* genotype without considering Aβ status. In 2018, phase III of this prevention trial, unfortunately, closed down because the results against symptomatic AD were negative, despite some improvement in brain metabolism. 

#### Dimebon

Another compound that affects mitochondria but failed the AD clinical trial was **dimebon** (**latrepirdine**). Dimebon (latrepirdine) is an old antihistaminic drug (first generation of H1-antagonist) used against allergies that was selected in an AD clinical trial because it demonstrated cognition and memory-enhancing properties in rats treated with neurotoxin (Bachurin et al., 2001). Moreover, dimebon showed a substantial effect on mitochondria from different AD models (**Table 1**). Anyway, dimebon lacked reproducibility in the AD clinical trials and showed opposite effects on neuropsychiatric and cognitive symptoms, and daily activities (Bachurin et al., 2001; Doody et al., 2008). In a review from 2018, Eckert et al. asked the scientific community to reevaluate the drug dimebon as a potential treatment of AD since one of the clinical trials was able to show a slight improvement of mitochondrial functions after using dimebon in respect of the substantial effect on cognition and behavior (Eckert et al., 2018).

## Conclusion

In a multitude of studies, mitochondrial dysfunction has been demonstrated to be a crucial feature of AD. Several experimental results suggested that a decline of mitochondrial activity happens during aging and may get worse at early stages of the disease, contributing to disease onset. However, more thorough investigations are needed to properly address this point. The suitability of the mitochondria as a target in AD treatment is still under discussion, considering that some pharmacological trials were not successful and others were more promising, but none led to a real marketable AD drug. Nevertheless, the current understanding of AD indicates that a complete cure may not be reachable yet. Future research efforts should be invested to i) understand the real chronology of events, ii) collocate correctly the mitochondrial dysfunction inside this temporal sequence, and iii) establish if the mitochondrial dysfunctions are a primary cause or a secondary event. Only when these three key points will be correctly settled, it will be easier to intervene pharmacologically and no more time and money will be wasted for futile therapeutic studies. The failures of the respective drugs or clinical trials often happened because the underlying scientific background was not always very robust or because the models and the tools used to prove the basal hypothesis were not always well defined or validated. Therefore, a more rational approach to a complex human disease like AD is needed as well as an improvement of communication between the different scientific disciplines in order to achieve a better understanding of the disease etiology and to develop new and more effective drugs.

## Author Contributions

GC conceived the idea and prepared the manuscript. WV reviewed the draft and provided important information for the completion of this manuscript.

## Funding

The Deutsche Forschungsgemeinschaft (Grant No VO 657/5-2 to WV) supported the work in our laboratory.

## Conflict of Interest Statement

The authors declare that the research was conducted in the absence of any commercial or financial relationships that could be construed as a potential conflict of interest.
